# Inconsistent trauma reporting is associated with emotional and behavioural problems and psychotic experiences in young people

**DOI:** 10.1186/s12888-020-2438-3

**Published:** 2020-01-31

**Authors:** Annette Burns, Helen Coughlan, Mary Cannon

**Affiliations:** Department of Psychiatry, Royal College of Surgeons in Ireland, Dublin 2, Ireland

**Keywords:** Adverse childhood experiences, Trauma, Trauma assessment, Consistency of reporting, Psychopathology, Psychotic experiences

## Abstract

**Background:**

Little is known about the prevalence of inconsistent trauma reporting in community samples and about its associations with psychopathology. This study aimed to assess for the first time the prevalence of inconsistent trauma reporting in a community sample of children/adolescents and to explore associations with both psychotic experiences and with psychopathology more generally.

**Method:**

A community-based sample of 86 children/adolescents (baseline mean age 11.5) were interviewed at two time points with data collected in relation to potentially traumatic events through the K-SADS. Emotional and behavioural problems were assessed at follow-up (mean age 15.7) through the Youth Self Report questionnaire while the presence of psychotic experiences was based on expert consensus post interview. Logistic regression models were used to test associations between inconsistent reporting and psychotic experiences at baseline and follow-up, with associations with emotional and behavioral problems at follow-up also assessed.

**Results:**

Overall, 16.3% of adolescents failed to report previously reported potentially traumatic events at follow-up and were therefore defined as inconsistent trauma reporters. Inconsistent reporting was associated with emotional and behavioural problems as assessed by the Youth Self Report with the exception of rule breaking behaviour and with psychotic experiences as assessed on interview.

**Conclusions:**

Inconsistent trauma reporting is associated with psychotic experiences and emotional and behavioural problems in young people and may represent an important marker for psychopathology in youth.

## Background

Research into the longitudinal consistency of reports of potentially traumatic events is limited and has traditionally focused on specific subgroups such as survivors of childhood sexual abuse [1] or military veterans [2, 3]. In 2006, Hepp et al. provided the first representative community-based study of consistency of reported exposure to potentially traumatic events over time by exploring this in a cohort study of young adults and found 20% failed to report trauma previously reported at a later time point while around a third reported potentially traumatic events for the first time during their second interview [4]. Inconsistent reporting in this manner was shown to be associated with higher self-esteem but unrelated to psychological functioning in terms of psychological problems, depression or mood syndromes in this community sample of young adults [4]. Associations with other psychological problems and psychosis or psychotic experiences were unexplored. More recently, Colman et al. explored consistency of reporting in a nationally representative cohort of adult Canadians and found 28.7% failed to report previously reported adverse childhood experiences and this inconsistency was associated with increased mastery, lower likelihood of psychological distress at baseline and lower likelihood of having developed depression, psychological distress or chronic stress between time points [5].

To date, while several papers explored trauma reporting in adults [4–6], no study has explored consistency of reporting of potentially traumatic events over time in a community sample of children/adolescents. The aim of the current study therefore was to assess the prevalence of inconsistent reporting of potentially traumatic events in a community sample of adolescents and to explore, for the first time in a child/adolescent sample, associations between inconsistent reporting and emotional and behavioral problems and for the first time in any sample associations between inconsistent reporting and psychotic experiences. The inclusion of a cognitive variable assessing memory as well as an item assessing quality of parental relationships in terms of tendency to disclose problems provided further novel data on associations with inconsistent trauma reporting.

## Methods

### Sample

**Fig. 1:**
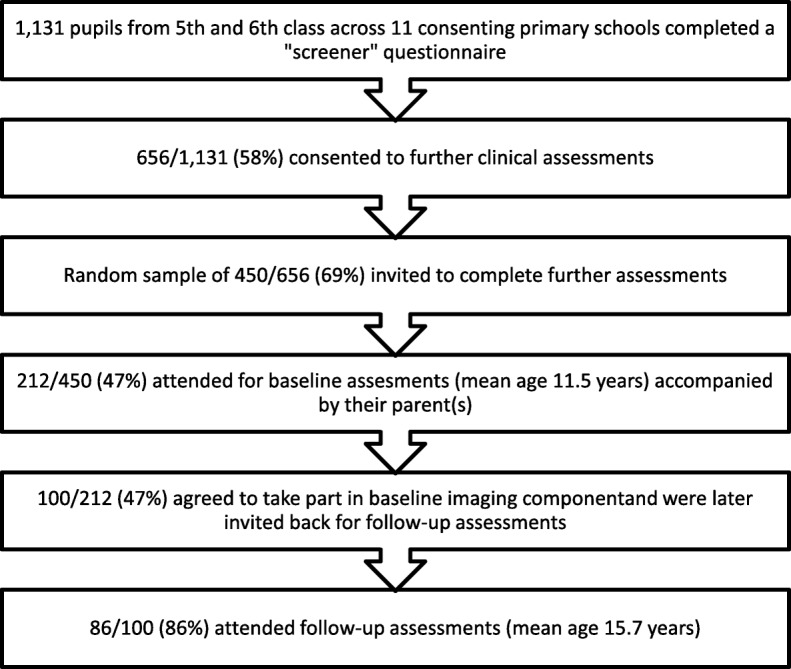
Adolescent Brain Development Study Recruitment

The initial screening sample for the Adolescent Brain Development Study (ABD) consisted of 1131 school children aged 11–13 years from 16 primary schools in Dublin, Ireland and neighboring counties who were screened using the Strengths and Difficulties Questionnaire (SDQ) [7] and the Adolescent Psychotic Symptom Screener (APSS) [8]. The recruitment process for the baseline sample has previously been reported in detail [8] but briefly, of the initial sample, 656 consented to taking part in the next stage of the study and from this a random sample of 450 were invited to assessments of which a subsample of 212 participants attended (See Fig. 1). Those attending for these further assessments (*n* = 212) did not significantly differ from the initial sample (*N* = 1131) [9].

At baseline, the full sample of 212 11–13 year olds (mean age 11.5) was assessed for mental disorders and neurocognitive functioning. Of those, 100 also participated in a brain imaging study [10]. These 100 were invited back to take part in a follow-up study (aged 14–16 years (mean 15.74)) and 86 agreed to take part. As previously reported, there were no significant differences in the baseline demographic and clinical characteristics of those attending follow-up assessments and those who did not in terms of gender, years in education, prevalence of psychotic experiences, mental disorder prevalence and functioning (CGAF and MSP-GAF scores). The only significant difference present was in relation to age, with participants attending follow-up assessments slightly older than those not attending follow-ups [11]. At follow-up, all participants again completed a clinical interview and a series of cognitive assessments. They also completed the Youth Self Report (YSR) [12]. At baseline and follow-up, all participants were asked questions about exposure to adverse and traumatic experiences as part of the clinical interview protocol.

### Measures

#### K-SADS-PL

The K-SADS-PL is an adapted version of the Schedule of Affective Disorders and Schizophrenia designed for children aged 6–18 years old [13]. It is a semi-structured diagnostic interview that assesses for all current (past month) and lifetime DSM Axis I mental disorders. ABD participants completed clinical K-SADS-PL interviews at each time point. At both time points they were assessed for DSM-IV Axis I disorders [14].

As part of the K-SADS-PL schedule, all participants were asked a series of 11 questions about potentially traumatic events as part the assessment for posttraumatic stress disorder. At both time points, individuals were asked to report any traumatic experience over the course of their lifetime. Participants were requested to report all potentially traumatic events that had ever happened, even if only once. Specifically, they were asked whether they had ever directly experienced 7 potentially traumatic events (a car accident, other accident, fire, violent crime, traumatic news, physical abuse, sexual abuse) and whether they had witnessed or been exposed to 3 potentially traumatic events (a disaster, a violent crime, or domestic violence). After asking about all these specific potentially traumatic events the interviewer also checked for any other potentially traumatic events by asking ‘Is there anything else that happened to you that was really bad, or something else you saw that was really scary, that you want to tell me about?’. At baseline both parents and children were interviewed separately in relation to the child’s experiences.

### Inconsistent trauma reporting

Responses were compared at baseline and follow-up for each of these potentially traumatic event questions. Following the exclusion of events reported by parents only at baseline, adolescents who failed to report at follow-up traumas they had previously reported were defined as ‘inconsistent trauma reporters’.

### Outcome measures

#### Psychotic experiences

Psychotic experiences were assessed using an enhanced version of the Psychosis section of the K-SADS-PL [13]. As part of the ABD study protocol, additional questions regarding attributions and distress related to reported hallucinatory and delusional experiences had been added to the Psychosis section of the K-SAD-PL. Participants were asked about any lifetime experiences of hallucinations and delusions. Hallucinations included auditory, visual, tactile, and olfactory hallucinations and participants were asked about the attribution of any phenomena reported. In relation to delusions, respondents were asked about unusual ideas and beliefs as well as persecution, grandiose, paranoid, somatic and nihilistic delusions. At baseline, both children and parents were interviewed about the child’s experiences while at follow-up reports came from adolescent participants only. An approach supported by the low concordance between adolescents and parents on experience of mental health [15].

At both time points, all interviewers had a background in psychiatry, psychology or mental health social work and were trained in the assessment of any reported psychotic phenomena. Detailed contemporaneous notes on all answers to questions about psychotic phenomena were recorded by the interviewer. These notes were saved as string data in the ABD study file. Following this, three mental health clinicians on the ABD study team reviewed all string data on potential psychotic experiences to determine rates of actual psychotic experiences within the sample while blind to all other information regarding the participants. Once each clinician completed his/her independent rating, a clinical consensus meeting was held where ratings were discussed and verified based on a set of criteria that had been developed for the ABD study. These criteria are summarised in Additional file 1.

#### Emotional and Behavioural difficulties

At follow-up, emotional and behavioural difficulties were measured using the Youth Self Report (YSR), a widely used 112-item self-report questionnaire designed to assess emotional and behavioural problems in 11–18 year olds [12]. Items are rated on a 3-point scale with 2 indicating the symptom is present most of the time, 1 indicating that the symptom is present some of the time or to some extent and 0 indicating the absence of the symptom. Scores are produced for total problems, YSR broadband scales (internalizing, externalizing), eight syndrome subscales (withdrawn, somatic, anxious/depressed, social problems, thought problems, attention problems, rule-breaking and aggressive behaviour) and social desirability. The YSR was administered at follow-up only.

#### Covariates/potential confounders

Associations for inconsistent trauma reporters compared to the rest of the sample were also assessed in relation to demographic variables and interviewer as well as follow-up variables assessing: memory (Wechsler Memory Scale which is part of the MATRICS cognitive battery [16]), functioning (Children’s Global Assessment Scale [17]), and whether they reported talking to their parents about their problems [assessed using a single-item self-report measure: ‘Would you talk to your parent(s) about a problem?’].

### Statistical analysis

Data were analysed using Stata 13.0. Descriptive statistics were used to profile the sample with Chi-square and t-tests used to compare those defined as inconsistent trauma reporters to the rest of the sample in terms of demographic information, YSR characteristics and potential confounders.

Logistic regression analysis was performed to model associations between inconsistent trauma reporting and psychotic experiences.

## Results

Responses were compared at baseline and follow-up for each potentially traumatic event question. Following the exclusion of events reported by parents only at baseline, 16.3% of young people failed to subsequently report a trauma they had reported at baseline at follow-up (*n* = 14) and were therefore defined as inconsistent trauma reporters.

Inconsistent trauma reporting was not significantly associated with age, gender, socioeconomic status (*χ*^*2*^ = 1.87, *p* = .867), interviewer (*χ*^*2*^ = 5.30, *p* = .151), class (in school) (*χ*^*2*^ = 3.93, *p* = .268), or current grade (*χ*^*2*^ = 4.13, *p* = .248) at baseline. In relation to follow-up variables, while inconsistent reporting was associated with highest year of education completed at follow-up (*χ*^2 =^12.7 *p* = .026), it was unrelated to age at follow-up, global functioning (CGAS), memory (WMS), social desirability or whether they reported talking to their parents about problems at follow-up (*χ*^*2*^ = 0.29, *p* = .589), a potential indicator of tendency to disclose or openness. As shown in Table 1, significant associations with inconsistent trauma reporting were found for all Youth Self-report (YSR) dimensions at follow-up, with rule breaking the only exception.

**Table 1:** Profile of sample including demographic information, psychotic experiences, Youth Self Report domains and potential confounders (*n* = 86)

	Rest of sample (*n* = 72)		Inconsistent reporters (*n* = 14)		t	*P* value
	M	SD	M	SD		
Age at Baseline (*n* = 76)	11.6	.58	11.5	.60	0.48	.630
	n	%	n	%	*χ*^*2*^	*P* value
Female	39	54.2%	7	50.0%	.082	.775
Psychotic Experiences Consensus at Baseline (n = 86)					4.19	.041*
None/Weak	59	81.9%	8	57.1%		
Definite	13	18.1%	6	42.9%		
Follow-up variables						
Talk to parents about problems (*n* = 77)	49	74.2%	9	81.8%	0.29	.589
Psychotic Experiences Consensus at Follow-up (n = 86)					10.0	.002**
None/Weak	65	90.3%	8	57.1%		
Definite	7	9.72%	6	42.9%		
Age at Follow-up (n = 86)	15.8	1.42	15.4	0.94	0.95	.344
Current functioning (CGAS) (*n* = 85)	83.4	10.8	81.1	11.7	.71	.475
Most severe past (CGAS) (n = 85)	72.1	15.1	69.4	13.2	.61	.546
Wechsler Memory Scale	50.6	10.7	46.8	3.99	1.32	.189
YSR total score	16.7	11.8	27.7	19.6	2.10	.041*
Syndrome subscales						
Withdrawn	2.62	2.09	4.92	3.42	3.15	.002**
Somatic Complaints	2.72	2.71	5.56	5.05	2.56	.013*
Anxious/Depressed	3.88	3.46	8	6.87	3.19	.002**
Social problems	2.95	2.88	5.15	4.14	2.33	.022*
Thought problems (includes hallucination/delusion items)	2.50	2.41	4.91	3.27	2.91	.005**
Attention problems	3.41	2.52	5.92	3.29	3.02	.003**
Rule breaking	3.40	2.91	4.33	3.45	.992	.324
Aggressive behaviour	4.41	4.44	7.50	5.47	2.13	.036*
Socially desirable items	21.9	3.04	21.4	3.70	.423	.674
Broadband dimensions						
Internalising	9.31	7.43	20.0	15.5	3.21	.002**
Externalising	7.93	7.11	12.8	7.93	2.06	.044*

** *P* < .01; *P < .05

### Inconsistent trauma reporting and psychotic experiences

As shown in Table 2, regression analysis revealed that inconsistent trauma reporting was significantly associated with psychotic experiences at follow-up (OR 6.96 95% CI [1.87–25.9] *p* = .004). This association also remained when psychotic experiences at baseline were added to the model (OR 6.91 95% CI [1.03–46.20) *p* = .046) indicating a significant prospective association (see Table 2).

**Table 2:** Logistic Models of Consensus Psychotic Experiences based on Inconsistent Trauma Reporting

				Adjusted for Psychotic Experiences at Baseline (None or weak/Definite)		
	Odds ratio	CIs	*P* value	Odds ratio	CIs	*P* value
Psychotic Experiences Consensus at Follow-up	6.96	1.87–25.9	.004**	6.91	1.03–46.20	.046*

** *P* < .01; *P < .05

## Discussion

This paper aimed to assess the prevalence of inconsistent trauma reporting in a community sample of children/adolescents and to assess its associations with psychopathology and psychotic experiences.

Overall, 16.3% of the sample reported a potentially traumatic event at baseline interview but failed to report the same potentially traumatic event at follow-up, and were therefore defined as inconsistent trauma reporters. This compares to prevalences of 20–28.7% for inconsistent reporting of the same nature in adult community-based [4] and population samples [5], 38–46% in studies of veterans [2, 3] and 50% in victims of child abuse [1].

In the current study, inconsistent trauma reporting was significantly associated with emotional and behavioural problems, as assessed by the YSR. Inconsistent reporters scored higher in terms of their total scores, scores in both broadband dimensions and their scores in 7 of 8 syndrome subscales (with rule breaking behaviour the only exception). Associations were strongest for internalising difficulties. These findings fit with previous work showing that lower self-disclosure is associated with poorer psychological functioning while disclosure of trauma appears beneficial for mental health, with evidence available for PTSD especially [18–20]. The lack of association found with rule breaking behaviour meanwhile, may be related to the fact that rule-breaking is said to peak in adolescence anyway [21].

Turning specifically to studies exploring consistency of trauma reporting over time however, Hepp et al. found largely no differences between consistent and inconsistent trauma reporters in their adult study, with no differences in terms of psychological problems, lifetime major depressive episodes, sub-threshold mood syndromes or depressive symptoms [4] while Colman et al. found omission at follow-up was actually associated with better outcomes in terms of stress, psychological distress and depression [5]. Notably, in their study of victims of abuse, Fergusson et al. also reported no associations between inconsistent reporting of childhood abuse and psychiatric state at follow-up [1]. The findings of the current study therefore suggest that this inconsistent reporting may be more concerning when occurring at an earlier age.

Indeed, the only significant group difference reported in Hepp et al.’s analysis of an adult community samples was in relation to self-esteem, which was higher in inconsistent reporters [4]. It should be noted however that this analysis employed a broader definition of inconsistent reporting including those who reported an earlier trauma for the first time at follow-up and it is possible results would have been different had they exclusively compared groups based on failure to subsequently report traumas reported at baseline as done in the current study. In Colman et al.’s population study, inconsistent reporters of this type also demonstrated increased mastery [5]. Unfortunately, the current study did not include assessment of mastery and thus its relevance in a younger sample of inconsistent trauma reporters currently remains unknown.

The focus of the current study on children and adolescents could also raise concerns regarding child memory. However, the ability of very young children to provide coherent, accurate and detailed reports of both routine and novel, one-time events has been established [22–25]. Moreover, in relation to traumatic events specifically, both clinical observations and large-scale investigation indicate that children form vivid memories which are retained over extensive delays [26] with studies of both natural disasters [27–30] and traumatic injuries [31, 32] demonstrating robust memory in children even after delays of several years.

Similarly, the possible suggestibility of younger children or tendency to over-report could be interpreted as a potential explanation for inconsistent reporting here. However, while studies show that preschool children are susceptible to suggestibility [33] older children have displayed high levels of concordance with parental reports in structured interviews, with agreement especially high for factual information (84%) compared to topics such as mental status (69%) [34].

Lower concordance on mental status or mental health between adolescents and parents on the other hand supports reliance on adolescent reports at follow-up [15, 34]. Waters et al. (2003) have found adolescent (aged 12–18 (mean age 15.1)) perceptions are significantly lower for their experience of mental health compared to their parents [15]. While in relation to psychotic experiences in particular, children and adolescents (aged 6–16) also appear to self-report hallucinations more frequently than their parents [35]. Thus, based on the available evidence in relation to memory, suggestibility and parent-child concordance, we believe that the participants in the ABD sample had the potential to be accurate reporters of recalled traumatic events at both baseline (mean age 11.5) and follow-up (mean age 15.7), as well as reliable self-reporters of their own mental health and the presence of any psychotic experiences at follow-up.

It has previously been suggested, that consistent reporting may indicate greater processing of an emotional experience, which in turn has been suggested to be related to successful adjustment [36, 37] and while in some studies no association between psychopathology and consistent reporting was found, an increased difficulty in reporting was still described [38].

Beyond adjustment, memory has also been put forward as an explanation for associations between recall and psychopathology or mood [39, 40]. For instance, research by Moore and Zoellner (2007) suggested that psychopathology may lead to overgenerality or difficulty retrieving specific memories [41]. Given experiencing traumatic events is known to increase risk of psychopathology, it may be, that in the current sample, traumatic events in childhood increase risk of psychopathology, which in turn increases overgenerality or risk of poorer recall in adolescence. This explanation is, however, challenged by the lack of association between memory scores and inconsistent reporting of potentially traumatic events in the current study, as well as other research, which has indicated there is little reason to link psychiatric status or poor mental health with less reliable or less valid recall of early experiences [42, 43]. For instance, in one study, Fogarty et al. showed that those with depression were in fact better at recalling sad memories compared to their non-depressed matched controls [44]. Moreover, in relation to inconsistent trauma reporting itself, Hepp et al. found self-reported memory problems were actually less frequent in inconsistent reporters [4]. Given the adolescent sample in the current study, it may also be that memory or recall bias were less of an issue with less time elapsed since the potentially traumatic events especially in comparison to the longer follow-up periods of up to 12 years observed in some studies [5].

Beyond psychopathology in general, experiencing traumatic events is also known to be associated with both psychosis [45, 46] and psychotic experiences [47, 48] and this has also been shown in young people [49] and community samples [50]. While the limited available data suggest that in a clinical population, patients with psychosis are reliable reporters of trauma [43], evidence in relation to community samples and associations with psychotic experiences rather than psychosis remains lacking. The small numbers in the current study did not allow for mediational analysis. However, given the significant associations with emotional and behavioural problems observed, and an internalising tendency in particular, further exploration of the potential mediating role of these issues in the relationship between inconsistent trauma reporting and psychotic experiences would be valuable. Particularly in light of the previously demonstrated associations between psychotic experiences and psychopathology in young people [51].

Other explanations of inconsistent trauma reporting include the natural degradation of memory over time [52, 53]. This theory does not however explain the particular associations with psychotic experiences and emotional and behavioural problems observed in the current study. Repression, a defence mechanism which is said to occur when the mind pushes some shocking experience into the unconscious [54], represents another possible interpretation of these findings. In their review of auditory verbal hallucinations, Longden and colleagues proposed these hallucinatory experiences could be conceptualised as unconscious dissociative responses to the experience of trauma [55]. Although our finding of an association between inconsistent reporting and psychotic experiences aligns to this psychodynamic interpretation, because the current study did not examine dissociation we were unable to examine this for the current study. With evidence of directed forgetting (active cognitive avoidance) and relabelling (reinterpreted as less upsetting or threatening [56, 57] within the available literature on trauma and abuse memories [58, 59], these also offer potential explanations for inconsistent trauma reporting. Thus, it may be that, over time, events experienced in childhood are no longer considered as severe or traumatic and thus were not discussed in adolescent interviews. It is also possible that some participants deliberately opted not to report a previously reported trauma at follow-up believing that only new traumas occurring since baselines assessment were of interest, even though, as noted above, all were asked to report lifetime events at each time point.

### Strengths

This is the first study, to our knowledge, to assess the prevalence of inconsistent reporting of potentially traumatic events in a community sample of children or adolescents. To our knowledge, it is also the first study to explore associations between inconsistent trauma reporting and psychotic experiences in a community sample. While the strong associations found require replication in other samples, further research may help to illuminate this potentially important relationship. The completion of a full clinical interview (K-SADS) [13] and follow-up consensus meeting of clinical experts (who were blinded to all other data) for each participant also means that the consensus on psychotic experiences in this study represents robust evidence in relation to the presence of hallucinations/delusional thinking at each time point.

Finally, the inclusion of a cognitive variable assessing memory, in the case of the Wechsler Memory Scale [16], as well as an item assessing quality of parental relationships in terms of tendency to disclose problems, provided novel data on associations with inconsistent trauma reporting not previously explored.

### Limitations

The current study assessed consistency of trauma reporting based on a checklist of potentially traumatic events and one open item. Arguably this approach therefore somewhat assumes these events were notable or traumatic and this may be a limitation when comparing to other literature on reported trauma. As such, there is a need replication of these findings in further community samples. In addition to the issue of generalisability, studies with larger samples would allow greater power to adjust for potential confounders when modelling associations between inconsistent trauma reporting and mental health as well as the potential to explore associations between specific types of psychotic experiences and the inconsistent reporting of trauma, which would be a valuable addition to the literature.

## Conclusions

Inconsistent trauma reporters in a child/adolescent sample were more likely to experience consensus-based psychotic experiences independent of the presence of these symptoms at baseline. They also evidenced greater emotional and behavioural problems, suggesting that, among adolescents inconsistent trauma reporting may be a marker for mental health problems.

## Supplementary information

**Additional file 1.** Summary of classification criteria for Psychotic Experiences for the Adolescent Brain Development Study

## Data Availability

The datasets used and analysed during the current study are available from the corresponding author on reasonable request.

## Abbreviation

ABD: The Adolescent Brain Development Study
