# Supplementary material for: Inconsistent trauma reporting is associated with emotional and behavioural problems and psychotic experiences in young people
Source: BMC Psychiatry. 2020 Jan 31;20:38. doi: 10.1186/s12888-020-2438-3 (PMC6993392; doi:10.1186/s12888-020-2438-3)
Supplement: Supplementary file 1 — Additional file 1. Summary of classification criteria for Psychotic Experiences for the Adolescent Brain Development Study [file 12888_2020_2438_MOESM1_ESM.docx]

Supplement 1

**Summary of classification criteria for Psychotic Experiences for the Adolescent Brain Development Study**

**Summary of rating and classification criteria**

All data reported in response to questions about hallucinatory or delusional experiences were rated according to the following categories:

**Definite Psychotic Experience:** The reported experience was definitely hallucinatory or delusional in nature

**Weak Psychotic Experience:** The reported experience had hallucinatory or delusional qualities but the perception, thought or belief was not strong or convincing enough to be classified as a definite psychotic experience

**Not a Psychotic Experience:** No evidence that the reported experience had any hallucinatory or delusion-like qualities

Participants who had at least one experience that was rated by consensus as being a Definite psychotic experience were classified as having experienced psychotic experiences for the study. Being rated as having a weak psychotic experiences only was insufficient to be classified as having psychotic experiences in this study. The table below gives examples of how experiences were defined for the purposes of rating.*

| HALLUCINATIONS |  |
| --- | --- |
| **Auditory Verbal Phenomena** | DEFINITE UPTB  Hearing one or more voices saying at least one word  Hearing whispering or indistinct voices at normal volume or shouting  Hearing name being called only if attributed to a delusional belief about another entity calling the name (e.g. a ghost)  Brief episodes of hearing own thoughts aloud when associated with delusional ideation and are either distressing or disorganising  WEAK UPTB  Brief episodes of hearing own thoughts aloud when not associated with delusional ideation and are neither distressing nor disorganising  NOT A UPTB  Hypnagogic and hypnopompic auditory verbal experiences |
| **Non-verbal Auditory Phenomena** | DEFINITE UPTB  Hearing non-verbal sounds that are experienced as distressing or disorganising  Hearing non-verbal sounds that are associated with delusional ideation  WEAK UPTB  Brief auditory experiences such as hearing music or other non-verbal sounds (e.g. hearing footsteps or knocking sounds) in the absence of any associated delusional ideation  NOT A UPTB  Hypnagogic and hypnopompic non-verbal auditory experiences |
| **Non-auditory Perceptual Phenomena** | DEFINITE UPTB  Seeing figures or forms that are not there (e.g. ghosts, human forms, aliens, the devil)  Tactile sensations that are associated with delusional attribution  WEAK UPTB  Recurrent experiences of smelling food that is not there  NOT A UPTB  Hypnagogic and hypnopompic visual experiences  Visual illusions  Occasional experiences of smells or tastes without any associated distress |
| DELUSIONS |  |
| **Unusual Thoughts and Beliefs** | DEFINITE UPTB  Delusional beliefs pertaining to any of the hallucinatory experiences in Domains 1-3  Definite and fixed beliefs about being watched by a person, entity or organisation  Recurrent and unfounded paranoid ideas that other people are criticising the individual  Beliefs that non-human entities (e.g. ghosts, spirits, aliens, the devil) are communicating directly with the individual  Unshakable nihilistic beliefs  Mind reading (self or others) when accompanied by paranoid beliefs that the individual has been singled out to have his/her mind read for a negative or nefarious purpose  WEAK UPTB  Vague sense or thought that individual is being watched  Mind reading (self or others) if not accompanied by a belief that the individual has been singled out to have his/her mind read for a negative or nefarious purpose  Magical thinking (e.g. a belief that the individual can predict the future) if not accompanied by distress or leading to disorganisation  NO UPTB  Thoughts and beliefs about being watched, judged or criticised by others that occur due to self-consciousness  A belief in ghosts, spirits or aliens are responsible or can influence experiences when such beliefs are aligned to normative cultural or subcultural beliefs  Subcultural beliefs that the world is coming to an end  Subcultural conspiracy beliefs |

* Exclusions: Hallucinatory experiences that occur in the context of an organic illness or that occur during acute intoxication

** Descriptions are not exhaustive
